# Effect of chlorocholine chlorid on phenolic acids accumulation and polyphenols formation of buckwheat plants

**DOI:** 10.1186/0717-6287-47-19

**Published:** 2014-05-27

**Authors:** Oksana Sytar, Asel Borankulova, Irene Hemmerich, Cornelia Rauh, Iryna Smetanska

**Affiliations:** Plant Physiology and Ecology Department, Taras Shevchenko National University of Kyiv, Institute of Biology, Volodymyrskya str., 64, Kyiv, 01033 Ukraine; Department of Technology of Food Products, Processing Industries and Biotechnology, Taraz State University named after MK Dulati, Suleimen Str., 7, Taraz, 080012 Republic of Kazakhstan; Department of Methods of Food Biotechnology, Berlin University of Technology, Institute of Food Technology and Food Chemistry, Koenigin Luise Str. 22, Berlin, D-14195 Germany; Agricultural Faculty, Department of Plant Food Processing, University of Applied Science Weihenstephan-Triesdorf, Steingruberstr. 2, Weidenbach, 91746 Germany

**Keywords:** Chlorocholine chloride, Phenolic acids, Catechins, Buckwheat

## Abstract

**Background:**

Effect of chlorocholine chloride (CCC) on phenolic acids composition and polyphenols accumulation in various anatomical parts (stems, leaves and inflorescences) of common buckwheat (*Fagopyrum esculentum* Moench) in the early stages of vegetation period were surveyed.

**Results:**

Treatment of buckwheat seeds with 2% of CCC has been increased content of total phenolics in the stems, leaves and inflorescences. On analyzing the different parts of buckwheat plants, 9 different phenolic acids – vanilic acid, ferulic acid, trans-ferulic acid, chlorogenic acid, salycilic acid, cinamic acid, *p*-coumaric acid, *p*-anisic acid, methoxycinamic acid and catechins were identified. The levels of identified phenolic acids varied not only significantly among the plant organs but also between early stages of vegetation period. Same changes as in contents of chlorogenic acid, ferulic acid, *trans*-ferulic acid were found for content of salycilic acid. The content of these phenolic acids has been significant increased under effect of 2% CCC treatment at the phase I (formation of buds) in the stems and at the phase II (beginning of flowering) in the leaves and then inflorescences respectively. The content of catechins as potential buckwheat antioxidants has been increased at the early stages of vegetation period after treatment with 2% CCC.

**Conclusions:**

The obtained results suggest that influence of CCC on the phenolics composition can be a result of various mechanisms of CCC uptake, transforming and/or its translocation in the buckwheat seedlings.

## Background

Buckwheat achene contain mostly carbohydrate, especially starch, 55,8% [1]. The starch content in buckwheat grains is 55,8%, in bran 40,7% and in the flour 78,4%. The protein content 11,7% in buckwheat grains, in bran 21,6% and in the flour 10,6% [1] Buckwheat protein content for different buckwheat species near 11-15% which is similar to the protein content in cereal grains. However, in cereals, 10-20% of the protein lies in the embryo, while 80-90% is found in the endosperm. In buckwheat 55% of the protein is located in embryo, 35% in the endosperm, and the reminder found in the hull [2]. Buckwheat can be grown in a nutrient-poor soil and requires only a short period from seeding to harvesting [3] and can be used as source for bread processing, especially development technology of using buckwheat seedlings with high phenolics content and high antioxidative capacities for bread processing.

In recent years, dietary plants such as buckwheat have attracted attention because they contain antioxidants that protect the human body from oxidative damage caused by free radicals. Buckwheat grain has a higher antioxidative activity than other cereal grains [4]; its antioxidative compounds include vitamins such as vitamins B1, B2 and E, as well as several phenolic compounds, which are found in the organs of buckwheat (leaf, stem and inflorescence) such as rutin, quercetin and proanthocyanidines (condensed tannins) [5, 6].

Tissues of buckwheat seedlings accumulate large concentration of various phenolic compounds [7]. In buckwheat seedlings has been found high content of chlorogenic acid [8] which is the most important cinnamic acid derivative [9]. In same time chlorogenic acid is the most potent functional inhibitor of the microsomal glucose-6-phosphate translocase (G6PT), is thought to possess cancer chemopreventive properties. It is also a promising precursor compound for the development of medicine that can resist AIDS virus HIV.

The phenolic compounds are important for plant due to their various biological functions including UV protection, pollen tube growth, antimicrobial activity, and insect resistance [10]. Simple phenolic acids such as *trans*-cinnamic and *p*-coumaric acids are precursors for more complex compounds including flavonoids, tannins, lignins and anthocyanins [10]. A series of naturally occurring phenolic acids with recognized anti-oxidant properties (derivatives of caffeic acid, rosmarinic acid, and trolox) have been conjugated with choline to account for the recognition by acetylcholinesterase. The synthesized hybrid compounds evidenced acetylcholinesterase inhibitory capacity of micromolar range (rationalized by molecular modeling studies) and good antioxidant properties and effects on human neuroblastoma cells for example [11].

CCC is an anti-gibberellin growth retardant. Treatment with 1.6 mM CCC resulted in the improved photosystem II (PSII) tolerance to UVB radiation, an increase in the contents of cytokinins, abscisic acid, and H_2_O_2_, which is one of molecule reactive oxygen species [12]. Exogenous CCC treatment has been found to improve crop performance under suboptimal growth conditions [13]; the high phenylalanine content in radish seedlings has been found under CCC treatment [14]. CCC controls the anthocyanin synthesis at the level of precursors so therefore it would be important for development of use CCC in the agriculture practice to study effect of CCC treatment on phenolic acids composition and their content in the plant crop such as buckwheat.

The changes of dynamics of total phenolics and phenolic acids formation in various anatomical parts (stems, leaves, inflorescences) of common buckwheat (*Fagopyrum esculentum* Moench.) in the early stage of vegetation period were surveyed what can be useful for development technology of growth buckwheat seedlings with high phenolics content and antioxidative capacities.

## Results and discussion

### Total phenolics

Phenolic compounds are plant metabolites characterized by the presence of several phenol groups. Some of them are very reactive in neutralizing free radicals by donating a hydrogen atom or an electron, chelating metal ions in aqueous solutions [15].

**Figure 1:**
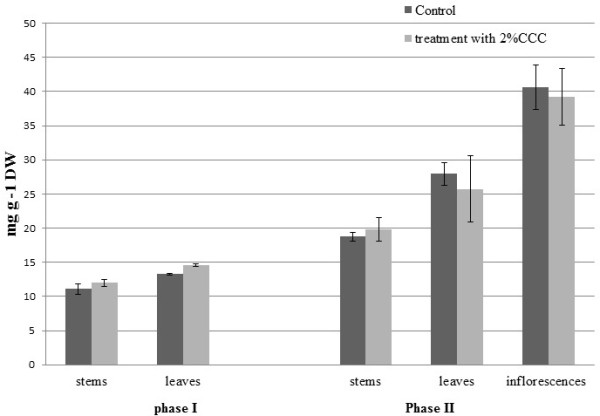
**Total phenolics content in buckwheat plants at the phase I (formation of buds) and phase II (at the beginning of flowering) treated with different 2% CCC.**

It was found that total phenolics content in leaves and stems of almond varieties changed according to season and plant organ [16]. In the variants with CCC treatment of buckwheat plants at phase I (formation of buds) it was visible tendency of increasing content of total phenolics in the leaves on 9% compared to the control, Figure 1.

In the phase II (at the beginning of flowering) in the stems of buckwheat plants has been observed tendency of increasing total phenolics content. Content of total phenolics in the leaves of buckwheat plants has been found tendency of increasing on 8% compared to the control. Content of total phenolics in the inflorescences in variant with CCC treatment was tendency of increasing on 8% compared to the control. Exogenous chlorocholine chloride (CCC) treatment has been found to improve crop performance under suboptimal growth conditions; however, the physiological mechanisms underlying the beneficial effects have not been fully understood. The treatment with certain concentration of CCC (e.g. 1.5–2.0 g L^-1^) improves mineral nutrition and superoxide dismutases, peroxidase and catalase activities in potato leaves; which might have contributed to the higher tuber yield of the crop grown under suboptimal conditions [13]. The increasing of superoxide dismutases, peroxidase and catalase activities can be connected with developing oxidative stress and increasing of reactive oxygen species (ROS) in the plant tissues under CCC treatment. We suppose that in this case is possible to expect increasing of total phenolic content in variants with CCC treatment as stress response reaction for neutralization of ROS. It was found that CCC promotes anthocyanin synthesis in radish plants at the early stages of growth. A higher amount of total free amino acids, in particular phenylalanine, was present in CCC-treated seedlings compared to controls grown on distilled water [14]. The first reaction in the phenylpropanoid pathway is catalyzed by phenylalanine ammonia-lyase (PAL; EC 4.3.1.5.) converting L-phenylalanine to *trans*-cinnamic acid [17]. It has been shown that *de novo* synthesis of phenylalanine ammonia-lyase isoforms is induced by biotic and abiotic elicitors [18, 19] and in a case with CCC it’s just confirmed increasing of total phenolic content in the buckwheat experimental variant in the early stages of vegetation period.

### Phenolic acids content and composition

On analyzing with HPLC the different parts of buckwheat plants, 9 different phenolic acids – vanilic acid, ferulic acid, *trans*-ferulic acid, chlorogenic acid, salycilic acid, cinamic acid, *p*-coumaric acid, *p*-anisic acid, methoxycinamic acid were identified (Tables 1 and 2).

**Table 1:** **Content of phenolic acids in the stems of buckwheat cultivar Rubra after treatment with 2% CCC**

	Stems			
	Phase I		Phase II	
	Control	2% CCC	Control	2% CCC
Vanilic acid	12,53 + 1,59	16,71 + 0,02*	15,21 + 0,75	38,21 + 8,96*
Chlorogenic acid	0,59 + 0,02	0,65 + 0,04*	1,53 + 0,14	3,25 + 0,35*
*p*-coumaric acid	8,79 + 1,67	9,55 + 0,96	9,73 + 0,15	21,10 + 5,60*
Ferulic acid	0,45 + 0,02	0,52 + 0,03*	0,68 + 0,05	7,83 + 1,20*
Trans-ferulic acid	19,62 + 3,45	21,89 + 2,35	49,01 + 7,87	59,82 + 2,29*
Salycilic acid	45,12 + 3,5	79,12 + 2,10*	59,78 + 2,16	360,39 + 49,22*
*p*-anisic acid	45,61 + 12,13	46,51 + 14,01	142,43 + 25,86	527,55 + 15,91*
Cinamic acid	4,02 + 1,23	5,10 + 1,32	4,34 + 0,23	8,71 + 1,55*
Methoxycinamic acid	5,36 + 0,81	5,74 + 0,96	6,76 + 0,63	43,16 + 4,46*

*Significant differences of these data were calculated using analysis of variance (ANOVA-Duncan’s multiple test, SIGMASTAT 9.0).

**Table 2:** **Content of phenolic acids in the leaves and inflorescences of buckwheat cultivar Rubra after treatment with 2% CCC**

	Leaves				Inflorescences	
	Phase I		Phase II		Phase II	
	Control	2% CCC	Control	2% CCC	Control	2% CCC
Vanilic acid	16,54 + 1,30	18,57 + 2,72	44,61 + 3,26	37,20 + 2,18*	27,95 + 1,06	77,93 + 7,39*
Chlorogenic acid	1,25 + 0,07	1,35 + 0,04*	3,27 + 0,35	3,63 + 0,67	1,52 + 0,04	3,06 + 0,85*
*p*-coumaric acid	17,54 + 0,22	19,25 + 0,32*	46,05 + 3,66	41,24 + 2,78	4,53 + 0,06	7,82 + 1,70*
Ferulic acid	2,35 + 0,43	3,89 + 0,15*	7,35 + 0,53	8,37 + 0,38*	1,81 + 0,14	2,24 + 0,76*
Trans-ferulic acid	35,31 + 3,48	45,03 + 4,24*	29,03 + 4,18	53,13 + 4,89*	20,12 + 2,36	28,87 + 6,56*
Salycilic acid	125,36 + 4,51	134,01 + 1,21*	166,01 + 4,40	164,50 + 5,01	295,25 + 2,57	367,01 + 41,02*
*p*-anisic acid	48,71 + 2,63	49,81 + 2,31	599,04 + 52,06	459,54 + 83,56*	612,13 + 12,5	817,01 + 43,05*
Cinamic acid	8,20 + 1,54	9,25 + 1,45	9,56 + 2,39	13,51 + 2,92*	8,02 + 1,23	8,99 + 2,51
Methoxycinamic acid	9,26 + 0,02	9,83 + 0,03*	19,10 + 6,70	17,51 + 1,08	10,82 + 1,23	11,74 + 2,10

*Significant differences of these data were calculated using analysis of variance (ANOVA-Duncan’s multiple test, SIGMASTAT 9.0).

The content of vanilic acid in the stems of buckwheat plants after treatment with 2% CCC has been increasing 25% and in the leaves at twice at the phase I (formation of buds). Then in the buckwheat leaves at phase II (at the beginning of flowering) has been shown decreasing of vanilic acid content on 16% compared to the control. Such decreasing can be connected with redistribution and increasing of vanilic acid content in the inflorescences as estimated content of vanilic acid there was higher more than 2 times compared to the control.

The effect of CCC also was visible in the changes of chlorogenic acid content in buckwheat plants at the early stages of vegetation period. It can evidence about stress response of buckwheat plants on CCC treatment as chlorogenic acid is an important antioxidant in plants, which can protects against lipid peroxidation [20]. At the phase I (formation of buds) in the stems content of chlorogenic acid has been increased on 9% and at the phase II (at the beginning of flowering) it was higher twice compared to the control. At the phase I (formation of buds) content of chlorogenic acid was significant higher in the leaves on 8% and in the inflorescences at 2 times compared to the control.

Major wall‒bound phenolics were 3,4‒dihydroxybenzoic acid, p‒coumaric acid and ferulic acid [21]. In the leaves at the phase I (formation of buds) content of *p*-coumaric acid has been shown tendency of increasing on 8% compared to the control. At the same time the content of *p*-coumaric acid in the inflorescences was higher on 42% compared to the control. At the phase II (beginning of flowering) the content of *p*-coumaric acid under the treatment with 2% CCC has been increasing in the stems on 54% compared to a control.

In the same time the increasing of *trans*-ferulic acid content in the leaves, stems and inflorescences of buckwheat plants at the phase I (formation of buds) and phase II (beginning of flowering) has been estimated. The content of *trans*-ferulic acid has been increased under effect of 2% CCC in the phase I (formation of buds) in the stems 18% and in the leaves 22% compared to the control variant. Increasing of *trans*-ferulic acid content at the phase II (beginning of flowering) in the leaves (45%) and in the inflorescences (30%) compared to control has been estimated.

The content of ferulic acid has been significant increase in the leaves at the phase I (formation of buds). In the phase II (beginning of flowering) ferulic acid has been significant increase in the stems on 18%, in the leaves on 45% and in the inflorescences on 30% respectively.

Cinnamic acid is one of the basic phenylpropanoid with antioxidant activity, produced by plants in response to stressful conditions. Exogenous cinnamic acid increased growth characteristics in saline and non-saline conditions in maize plants. But effects of cinamic acid were more significant under saline conditions in comparison to non-saline conditions [22]. Cinamic acid relatively increased the leaf relative water content and the chlorophyll content, decreased plasma membrane permeability, mitigated membrane damage, inhibited the accumulation of malondialdehyde (product of membrane lipid peroxidation), and promoted the activity of membrane protective enzymes such as super oxide dismutase and peroxidase [23]. In same time cinnamic acid is a precursor in biosynthetic pathway of salicylic acid signaling molecule [24].

The content of cinamic acid during the phase I (formation of buds) in the stems, leaves was on control level. At the phase II (beginning of flowering) in the leaves has been found increasing of cinamic acid content on 29% and in the stems at twice compared to a control variant. In the inflorescences at the phase II (beginning of flowering) content of cinamic acid was on control level.

In higher plants, it is well established that salicylic acid derives from the shikimate-phenylpropanoid pathway [25]. Currently, it has been reported that this compound plays also a role in plants responses to abiotic stresses, such as drought, low and high temperatures, heavy metals, and osmotic stress [26–30]. Salycilic acid was also shown to influence a number of physiological processes, including seed germination, seedling growth, fruit ripening, flowering, ion uptake and transport, photosynthesis rate, stomata conductance, biogenesis of chloroplast [31–33].

Same changes as in content of other identified phenolic acids (chlorogenic acid, ferulic acid, *trans*-ferulic acid) were found for content of salycilic acid. The changes with increasing salycilic acid content has been observed in the buckwheat plants. The content of salycilic acid in phase I and phase II in the different part of buckwheat plants has been increased. The content of salycilic acid in the phase II (beginning of flowering) for inflorescences has been increased on 20%, for stems – on 80%.

It was estimated that metabolic pathway of salicylic acid rather than of chlorogenic acid is involved in the stress-induced flowering of *Pharbitis nil* (Japanese morning glory) plants [34]. The metabolic pathway from *t*-cinnamic acid to salycilic acid via benzoic acid is involved in the stress-induced flowering which can confirm also significant increasing of salicylic acid content in the stems of buckwheat plants in the phase II (beginning of flowering) compared to the phase I (formation of buds). At the same time significant increasing content of salycilic acid in the leaves and inflorescences in variant with CCC treatment in the phase II (beginning of flowering) is evidence about role of salycilic acid under plant stress conditions which could be occurred CCC treatment. Salycilic acid is an endogenous regulator of growth involved in a broad range of physiologic, metabolic and stress responses in plants [35].

At the phase II (beginning of flowering) has been observed increasing of *p*-anisic acid content in the stems on 73% and in the inflorescences 25% compared to the control. In the leaves at the phase II (beginning of flowering) has been shown significant decreasing of *p*-anisic acid content (23%). Same decreasing of vanilic acid content in the phase II (beginning of flowering) has been estimated. Such decreasing can be connected with redistribution and increasing of content these phenolic acids in the inflorescences compared to the control.

At phase I (formation of buds) content of methoxycinamic acid has been increased in the leaves of buckwheat on 6% compared to the control. During the phase II (beginning of flowering) has been shown significant increasing of methoxycinamic acid on 84% in the stems of buckwheat plants.

### Catechins content

**Figure 2:**
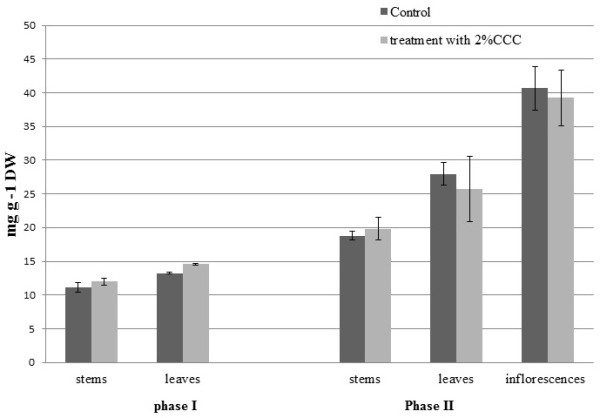
**Content of catechins in the buckwheat plants of different plant parts after treatment with 2% CCC.**

On analyzing with HPLC the different parts of buckwheat plants catechins has been estimated (Figure 2).

In the stems of buckwheat plants at phase II (beginning of flowering) in variant with CCC treatment has been shown catechins content higher 67% compared to the control. In the leaves have been estimated increasing of catechins content 32% compared to the control. In the inflorescences of buckwheat plants under CCC treatment content of catechins has been increased more than twice.

Catechins are a type of antioxidant found in the greatest abundance in the leaves of the tea plant *Camellia sinensis*. In smaller amounts, they are found in other foods such as wine, chocolate, berries, and apples. Their health benefits of have been under close examination since the 1990s, due to the strong association of tea with long life and health in many ancient cultures [36].

Watanabe has been identified 4 catechins in the buckwheat (*Fagopyrum esculentum* Moench) groats. The structures of these catechins were established as (-)-epicatechin, (+)-catechin 7-O-D-glucopyranoside, (-)-epicatechin 3-O-p-hydroxybenzoate, and (-)-epicatechin 3-O-(3,4-di-O-methyl) gallate on the basis of 1H, 13C. The antioxidant activity of the isolated compounds showed that the activity of catechins was superior to that of rutin, which is known as an antioxidant in buckwheat, at the same concentration [6].

The content of catechins as potential buckwheat antioxidants has been increased at the early stages of vegetation period after treatment with 2% CCC. It’s known that catechins can be potential antioxidants among phenolic compounds.

## Conclusions

It’s known that vegetative mass of buckwheat plants is not used in food industry well and vegetative organs (leaves, stems and inflorescences) can contain higher antioxidants composition than buckwheat seeds. Therefore to find way of increasing content of buckwheat antioxidants is actual topic nowadays. In this research work we suggested simple idea to use chlorocholine сhloride as factor which can increase content of phenolic compounds. The obtained results suggest that influence of CCC on the phenolics composition can be a result of various mechanisms of CCC uptake, transforming and/or its translocation in the buckwheat seedlings. The levels of identified phenolic acids varied not only significantly among the plant organs but also between early stages of vegetation period. Same changes as in contents of chlorogenic acid, ferulic acid, *trans*-ferulic acid were found for content of salycilic acid. The content of these phenolic acids has been significant increased under effect of 2% CCC treatment at the phase I (formation of buds) in the stems and at the phase II (beginning of flowering) in the leaves and then inflorescences respectively. The enhanced accumulation of total phenolics, catechins and different changes for free phenolic acids (especially increasing content of chlorogenic acid and salycilic acid) can be explained probably by the synthesis of other unknown phenolic compounds or role some phenolic acids in the stress response reaction which could cause CCC treatment.

## Methods

The common buckwheat (*Fagopyrum esculentum* Moench) cultivar Rubra has been used for this experimental work. Cultivar Rubra with high anthocyanins content 3.87 - 4.41 mg/100 g DW in the vegetative organ has been received by family selection method from chemo mutants from Taras Shevchenko National University of Kyiv.

Seeds were germinated between two layers of wet filter paper, which then were rolled and inserted in a 24 beaker containing 200 ml of tap water (Control) and solution of 2% CCC for 4 days (Experiment). Germination process was carried on in the darkness at 24 ± 1°C. After 4 days grown in such conditions the seedlings of buckwheat were taken to growth in the pots in 16/8 h night/day photoperiod and 65 ± 5% of relative humidity. Temperature in growth chamber was maintained at 24 ± 2°C for day and 18 ± 2°C during night period. The content of total phenols and phenolic acids has been evaluated in growth phase I (formation of buds), in phase II (at the beginning of flowering).

### Determination of total phenolics

Total phenolics were determined by using Folin-Ciocalteu reagent [37]. 0.02 g powdered samples (freeze-dried) were extracted for 10 min with 500 mL of 70% methanol at 70°C. The mixtures were centrifuged at 3500 g for 10 min and the super-natants were collected in separate tubes. The pellets were re-extracted under identical conditions. Supernatants were combined and used for total phenolics assay and for HPLC analysis. For total phenolics assay 20 mL of extract was dissolved into 2 mL of distilled water. Two hundred microliters of dissolved extract were mixed with 1 mL of Folin-Ciocalteu reagent (previously diluted tenfold with distilled water) and kept at 25°C for 3–8 min; 0.8 mL of sodium bicarbonate (75 g L^-1^) solution was added to the mixture. After 60 min at 25°C, absorbance was measured at 765 nm. The results were expressed as gallic acid equivalents.

### HPLC analyses of flavanols and phenolics acids

The plant material was harvested and frozen in liquid nitrogen for the preventing of phenolic compound volatilization. Afterwards the samples were lyophilized. Further, finishing the freeze-drying process the material was grounded by flint mill (20000 g, 2 min). A total of 20 mg grounded samples from leaves suspension were extracted for 15 min using 0.75 mL 70% methanol (v/v, pH 4.0, phosphoric acid) in ultrasonic water bath on ice. Samples were centrifuged for 5 min at 6000 g. The supernatants were collected and the pellets were re-extracted twice more with 0.5 mL 70% methanol. Coumaric acid or cinnamic acid (40 mL of 3 mM solution) was added as internal standard to the first extraction. The combined supernatants from each sample were reduced to near dryness in a centrifugation evaporator (Speed Vac, SC 110) at 25°C.

Samples were added up to 1 mL with 40% acetonitrile. The samples were filtrated using 0.22 mm filters, and then analyzed with HPLC. The chromatography was performed using a Dionex UltiMate 3000 HPLC System with a diode array detector (DAD-3000) with a WPS-3000 SL auto sampler, LPG-3400SD pump and a TCC-3000RS Column Compartment (Dionex Corp., Sunnyvale, CA, USA).

Extracts (1 mL) were analyzed at a flow rate of 0.4 mL 1 min and a column temperature of 35°C. The column is Narrow-Bore Acclaim PA C16-column (3 mm, 120A, 2.1 × 150 mm, Dionex). A 49-min gradient program was used with 0,1% v/v phosphoric acid in ultrapure water (eluent A) and of 40% v/v acetonitrile in ultrapure water (eluent B) as follows: 0–5 min: 0.5% B, 1–9 min: 0–40% B, 9–12 min: 40% B, 12–17 min: 40–80% B, 17–20 min: 80% B, 20–24 min: 80–99% B, 24–32 min: 99–100% B, 32–36 min: 100–40% B, 36–49 min: 40–1% B. The gradient program was followed by a 4 min period to return to 0.5% B and a 5 min equilibration period resulting in a total duration of 39 min. The eluent was monitored at 290, 330, and 254 nm.

### Statistical analysis

The means and standard deviations were calculated by the Microsoft Office Excel 2003. Significant differences of these data were calculated using analysis of variance (ANOVA-Duncan’s multiple test, SIGMASTAT 9.0). All results were expressed as mean ± standard deviations from three and four replications.

## Availability of supporting data

The data sets supporting the results of this article are included within the article.

## Disclosure statement

No competing financial interests exist.

## Abbreviations

CCC: Chlorocholine chloride
ROS: Reactive oxygen species
HPLC: High-performance liquid chromatography.

## References

[1] Bonafaccia G, Marocchini M, Kreft I (2003). Composition and technological properties of the flour and bran from common and tartary buckwheat. Food Chem.

[2] Aufhammer W (2003). Pseudogetreidearten – Buchweizen, Reismelde und Amarant; Herkunft, Nutzung und Anbau. J Agr Crop Sci.

[3] Ikeda K (2002). Buckwheat: composition, chemistry and processing. Adv Food Nutr Res.

[4] Zielinski H, Kozlowska H (2000). Antioxidant activity and total phenolics in selected cereal grains and their different morphological fractions. J Agric Food Chem.

[5] Watanabe M, Ohshi Y, Tsushida T (1997). Antioxidant compounds from buckwheat (*Fagopyrum esculentum* Moench) hulls. J Agric Food Chem.

[6] Watanabe M (1998). Catechins as antioxidants from buckwheat (*Fagopyrum esculentum* Moench) groats. J Agric Food Chem.

[7] Kim HJ, Park KJ, Lim JH (2011). Metabolomic analysis of phenolic compounds in buckwheat (*Fagopyrum esculentum* M.) sprouts treated with methyl jasmonate. J Agric Food Chem.

[8] Sytar O, Zhenzhen C, Brestic M, Prasad MNV, Taran N, Smetanska I (2013). Foliar applied nickel on buckwheat (*Fagopyrum esculentu*m) induced phenolic compounds as potential antioxidants. Clean - Soil, Air, Water.

[9] Hahlbrock K, Scheel D (1989). Physiology and molecular biology of phenylpropanoid metabolism. Annu Rev Plant Physiol Plant Mol Biol.

[10] Winkel-Shirley B (2002). Biosynthesis of flavonoids and effects of stress. Curr Opin Plant Biol.

[11] Šebestík О, Marques SM, Falé PL, Santos S, Arduíno DM, Cardoso SM, Oliveira CR, Serralheiro MLM, Santos MA (2011). Bifunctional phenolic-choline conjugates as anti-oxidants and acetylcholinesterase inhibitors. J Enzyme Inhib Med Chem.

[12] Kreslavskiia VD, Lubimova VY, Kotova LM, Kotov AA (2011). Effect of common bean seedling pretreatment with chlorocholine chloride on photosystem II tolerance to UVB radiation, phytohormone content, and hydrogen peroxide content. Russ J Plant Physiol.

[13] Huiqun W, Langtao X, Jianhua T, Fulai L (2010). Foliar application of chlorocholine chloride improves leaf mineral nutrition, antioxidant enzyme activity, and tuber yield of potato (*Solanum tuberosum* L.). Scient Horticul.

[14] Jain VK, Guruprasad KN (1989). Effect of chlorocholine chloride and gibberellic acid on the anthocyanin synthesis in radish seedlings. Physiol Plant.

[15] Petti S, Scully C (2009). Polyphenols, oral health and disease: A review. J Dent.

[16] Sivaci A, Duman S (2014). Evaluation of seasonal antioxidant activity and total phenolic compounds in stems and leaves of some almond (*Prunus amygdalus* L.) varieties. Biol Res.

[17] Hanson KR, Havir EA, Stumpf PK, Conn EE (1981). Phenylalanine ammonia-lyase. The biochemistry of plants.

[18] Jahnen W, Hahlbrock K (1988). Differential regulation and tissue-specific distribution of enzymes of phenylpropanoid pathways in developing parsley seedlings. Planta.

[19] Schmelzer E, Jahnen W, Hahlbrock K (1988). In situ localization of light-induced chalcone synthase mRNA, chalcone synthase, and flavonoid products in epidermal cells of parsley leaves. Proc Natl Acad Sci U S A.

[20] Niggeweg R, Michael AJ, Martin C (2004). Engineering plants with increased levels of antioxidant chlorogenic acid. Nat Biotechnol.

[21] Schützendübel A, Polle A (2001). Plant responses to abiotic stresses: heavy metal‒induced oxidative stress and protection by mycorrhization. J Exp Bot.

[22] Pramod KS, Ramendra S, Shivani S (2013). Cinnamic acid induced changes in reactive oxygen species scavenging enzymes and protein profile in maize (*Zea mays* L.) plants grown under salt stress. Annu Rev Plant Biol.

[23] Xuezheng W, Hua W, Fengzhi W, Bo L (2007). Effects of cinnamic acid on the physiological characteristics of cucumber seedlings under salt stress. Front Agric China.

[24] Hayat Q, Hayat S, Irfan M, Ahmad A (2010). Effect of exogenous salicylic acid under changing environment: A review. Environ Exp Bot.

[25] Stitcher L, Mauch-Mani B, Metraux JP (1997). Systemic acquired resistance. Annu Rev Plant Pathol.

[26] Molina A, Bueno P, Marín MC, Rodríguez-Rosales MP, Belver A, Venema K (2002). Involvement of endogenous salicylic acid content, lipoxygenase and antioxidant enzyme activities in the response of tomato cell suspension cultures to NaCl. New Phytol.

[27] Nemeth M, Janda T, Horvath E, Paldi E, Szalai G (2002). Exogenous salicylic acid increases polyamine content but may decrease drought tolerance in maize. Plant Sci.

[28] Munne-Bosch S, Peñuelas J (2003). Photo- and antioxidative protection, and a role for salicylic acid during drought and recovery in field-grown *Phillyrea angustifolia* plants. Planta.

[29] Shi Q, Zhu Z (2008). Effects of exogenous salicylic acid on manganese toxicity, element contents and antioxidative system in cucumber. Environ Exper Bot.

[30] Rivas-San Vicente M, Plasencia J (2011). Salicylic acid beyond defence: its role in plant growth and development. J Exp Bot.

[31] Fariduddin Q, Hayat S, Ahmad A (2003). Salicylic acid influences net photosynthetic rate, carboxylation efficiency, nitrate reductase activity and seed yield in *Brassica juncea*. Photosynthetica.

[32] Khodary SFA (2004). Effect of salicylic acid on the growth, photosynthesis and carbohydrate metabolism in salt stressed maize plants. Int J Agric Biol.

[33] Hayat S, Fariduddin Q, Ali B, Ahmad A (2005). Effect of salicylic acid on growth and enzyme activities of wheat seedlings. Acta Agron Hung.

[34] Hatayama T, Takeno K (2003). The metabolic pathway of salicylic acid rather than of chlorogenic acid is involved in the stress-induced flowering of *Pharbitis* nil. J Plant Physiol.

[35] Hayata Q, Hayata S, Irfana M, Ahmad A (2010). Effect of exogenous salicylic acid under changing environment: a review. Environ Exp Bot.

[36] Sytar O, Brestic M, Rai M, Shao HB (2012). Plant phenolic compounds for food, pharmaceutical and cosmetiсs production. J Med Plants Res.

[37] Singleton VL, Rossi JA (1965). Colorimetry of total phenolics with phosphomolybdic-phosphotungstic acid reagents. Am J Enol Vitic.

